# Unrestricted prevalence of sedentary behaviors from early childhood

**DOI:** 10.1186/s12889-020-8346-0

**Published:** 2020-02-19

**Authors:** Fariba Azabdaftari, Parisa Jafarpour, Mohammad Asghari-Jafarabadi, Behjat Shokrvash, Parvin Reyhani

**Affiliations:** 1 Basic Sciences Department, Paramedical School, Tabriz University of Medical Sciences, University Campus, Danshgah Street, Tabriz, Iran; 2 Department of Health Education and Promotion, Faculty of Health, Tabriz University of Medical Sciences, Golgasht Street, Tabriz, Iran; 3 RoadTrafc Injury Research Center, Tabriz University of Medical Sciences, Tabriz, Iran; 4 Department of Statistics and Epidemiology, Faculty of Health, Tabriz University of Medical Sciences, Tabriz, Iran; 5 Medical Education Research Center, Health Management and Safety Promotion Research Institute, Tabriz University of Medical Sciences, Golgasht Ave, Tabriz, Iran

**Keywords:** Sedentary behavior, Television watching, Electronic media communication, Computer use, Child, Parent, Knowledge, Communicative style, Family affluence scale, Socio-economic status

## Abstract

**Background:**

Light and sedentary behaviors impose heavy challenges on societies. The objectives of this study are to identify child sedentary behaviors, and to examine the relationship between parent knowledge and behavioral style on children’s sedentary time in Iran.

**Methods:**

This cross-sectional study was done among children and their parents selected randomly using multi-stage method, from 12 urban districts in Tabriz, Iran;2017. Data were collected through designing a multi-sectional questionnaire adopted from the Bjelland and previous studies to assess the time spent on sedentary behaviors among children/adolescents along with parent knowledge and behavioral style.

**Results:**

From 480 children/adolescents and their parents 54.6% came from middle class families, and 55.62% were boys aged 2 to18. The percentage of time spent more than 120 min per day (min/d) on weekdays was for watching television (TV): (girls 24.4%, boys 21.0%), for playing computer and video games: (girls 38.7%, boys 54.7%), for electronic media communication (EMC): (girls 52.8%, boys 60.2%). The associated factors for watching TV: child age [12 years and above OR = 1.37; 95% CI = 0.53–3.54], parent knowledge [OR = 0.59, 95% CI = 0.35–0.99], and communicative styles [OR = 1.43, 95%CI = 1.11–1.86], and for playing computer and EMC: child age [5 years old and above OR = 4.83,95% CI =1.52–15.38, 12 years old and above OR = 13.76, 95% CI= 4.22–24.91], family socio-economic status [middle class OR = 2.52, 95% CI = 1.54–4.11, high class OR = 5.53, 95%CI = 1.80–15.89].

**Conclusion:**

There is an urgent need to combat the unrestricted prevalence of sedentary behaviors among Iranian children/ adolescents who use computers and other electronic devices more than the recommended time every day from early childhood.

Parents should be provided with appropriate information about adverse effects of using electronic devices longer than recommended time by children. It is also essential to teach them beneficial communicative styles to monitor their children’s sedentary behaviors.

## Background

Sedentary behaviors as light activities [1] impose adverse consequences on population in general, and on children/ [2–5] adolescents in particular [6–9]. Sedentary behaviors cover wide ranges of activities such as watching TV, playing video games, working on computer, talking on the telephone, studying, sitting while doing homework, and other sitting activities [1, 10–12], which require minimal energy [1, 5].

According to the advice of pediatric professionals children over 7 years old should not be involved in sedentary activities for more than 120 min a day [6, 11]. However, children/ adolescents spend more than 120 min/d on sedentary behaviors all over the world [12–14]. A recent study in Eastern Mediterranean Region countries shows a high prevalence of sedentary behaviors among children/adolescents [15].

Personal, family, and environmental factors affect the pattern and duration of sedentary behaviors among children/adolescents [16]. The most crucial factors are child gender and age [13, 17, 18], environment [19–21], family socio-economic status [17, 18, 22], parent characteristics [23], along with their behavioral [24–26] and communicative styles [27, 28].

Parental styles are the primary predictors of watching TV among preschoolers on weekdays and on the weekend [25, 26]. The Bjelland et al. study in five European countries showed that supportive-authoritative parents and setting regulations had a significant relationship with watching TV and playing computer games, as increases in authoritative parenting reduced the time children spent watching TV and playing computer games [27]. Supportive, communicative and authoritative parents encourage self-awareness in their children [28].

Further research suggests to identify both possible and actual reasons for the ever growing rate of sedentary behaviors among children/adolescents [1, 6, 12, 23]. In their systematic review study, Rollo et al. [23] suggested more in-depth research to comprehend the relationship between cognitive and motivational factors affecting the duration of sedentary behaviors.

In Iran several studies have been done on the patterns of physical activities among children [29–31] and associated factors such as the role of personal, family and social status, body mass index, and sleep patterns [32]. However, no evidence regarding the relationship between parent knowledge and communicative style with child sedentary behavior was found. The objectives of this study were to identify child sedentary behaviors, to examine the relationship between family socio-economic status along with parent knowledge and behavioral style on children’s sedentary time in Iran.

## Methods

### Study design and study participants

This cross-sectional study was carried out among 480 children/ adolescents from 2 to 18 years old and their parents who were covered by the health centers of Tabriz City Iran. The recruitment of the participants and the collection of data were carried out during Apr-Jul 2017. Samples were selected randomly using a multi-stage method, from the 88 rural health centers of the Tabriz area. The sample size was calculated based upon previously identified data regarding the relationship between sedentary behavior and demographic variables (OR = 2.36) [29]. Considering 95% confidence level, 80% power, a two-tailed test and utilizing the PASS15 software, the sample size was estimated to be 240 cases. Taking into account the sampling design effect of two, the required sample size increased to 480 cases. At the time of study there were 88 urban active health centers in Tabriz city. Firstly, 30% of the urban health centers (26.4), in all 27 urban health centers were selected as study sample. Secondly, samples (parents, children) were randomly selected according to the sample size estimation (480) and the rate of population covered by each center. The samples were taken systematically based on the inclusion criteria (being residence of the Tabriz area, being interested in participating in the study, not having any physical and /or mental disorders), and the list of households.

All activities were harmonized and coordinated with the health center authorities. Invitations were sent to participants to take part in the study on a designated day. After stating the study goals and the process of participation, the participants were asked to fill in the questionnaires.

### Data collection and measures

Data collection was performed by using several questionnaires; a demographic questionnaire consisted of the child sex and age, parent education and occupation, and family socio-economic status. The child age grouping was categorized as 2–4, 5–11, and 12 and above. The mother age grouping also was categorized as under 34, 35–44, 45–54, and 55 and above.

Socio-economic status of the families were determined by the valid family affluence scale (FAS) [33, 34]. It contained 14 common household properties: private bedroom, laundry, dishwasher, TV set, personal computer (PC), laptop, tablet, landline telephone, smartphone, and access to internet network. Answers were categorized into two levels: don’t have/not existing = 0 and have/existing = 1, and more. The scores ranged from 8 to 60, and were categorized into three levels of FAS: low = 8–25, medium = 26–42, and high = 43 and above.

The pattern of parent behavioral style in the present study was taken from Bjelland study [27], following two major variables: a. setting regulations, b. communicative style (b.1.threats and Punishment; b.2.logical communication and explaining reasons). Overall nine questions were used to assess parent style, three items for setting regulations, to control the time spent on watching TV, using computers and playing video games as well as smartphone. Six items were given for parent communicative style, to make children follow regulations through two ways of either punishment and threatening or logical style and explaining reasons. The answers were prepared based on likert scale (strongly agree = 5, agree = 4, no idea = 3, disagree = 2, strongly disagree = 1). The possible range for each scale was 3 to15. The higher score meant the higher degree of the parent behavioral style. That is, parents reported more regulations and more logical reasoning. A cut-off of 12 was used for determining ‘setting regulation, and applying a punishment style’s scores into two levels. Scores below 12 were considered to as not setting the regulation, or did not apply a punishment style, and 12 and above as setting the regulation or applied reasoning.

The guideline of recommended time for sedentary activities [12,35] was an indicator of assessing parent knowledge. Parent knowledge was measured using three questions: how much time experts recommend children spend on sedentary activities; under 2 years, 2–4 years, and 5 years and above. The answers were categorized based on six scales: not at all, 30 min/d, 60 min/d, 90 min/d, 120, and above. The parent answers for each age group recorded in two levels of ‘know’ and ‘don’t know’. For age groups: (under2), (2–4), and (5 and above), the correct answers were: (not at all), (less than 1 h/d), and (less than 2 h/d) respectively.

The sedentary behavior questionnaire was prepared based on existing studies [12, 29] to evaluate the frequency and time period of 10 sedentary behaviors during a week. Time period of each sedentary behavior listed according to min and hours (h) spent for each activity following the below pattern: 0 min, 30 min, 60 min (1 h), 90 min (1 h and 30 min), and so on. The sedentary behaviors of children/ adolescents were the mean period of sitting time (min/d) included watching TV and playing video games, working on the computer, playing games on computers, doing homework, studying, listening to music while lying down, day time sleep, and night time sleep on weekdays and on the weekend. The total time of the activities were estimated first included and then excluded the amount of night time sleep.

Activities such as watching TV, playing video and computer games, studying, and EMC were estimated individually. EMC included time spent on playing games on smartphone, talking on the phone, sending message, photo, file, and virtual communication. The time of activities estimated based on the guideline recommending < 120 min/d [10, 12, 35].

The self-administrated questionnaire was used for data collection from literate parents and children 10 years old and above, while for illiterate parents and children under 10 years old the questionnaire was filled in by the interviewer.

### Statistical analysis methods

Descriptive statistic methods, frequency and percentages for qualitative variables and mean (M) and standard deviation (SD) for quantitative variables, were used. To determine statistical differences between groups, chi-square and independent t-tests were used.

Multivariate logistic regression analysis was used to determine the association between dependent variables (outcomes) and independent variables (predictors). Dependent variables were watching TV, playing computer games, communicating electronic media, and studying duration. Independent variables (predictors) were parent behavioral style, parent knowledge and FAS. The potential controlling variables were child sex, age, mother education, age, and job.

Analyzing data was performed individually for each dependent variable. Each dependent variable was divided based on time spent according to min/d: the recommended time under 120 min/d, recoded as 0; and 120 min/ and above considered as high risk time recoded as 1. Analyzing data was performed using SPSS 21, *P*-value lower than 0.05. The study was reported according to the ‘Strengthening the Reporting of Observational Studies in Epidemiology (STROBE) guidelines’.

## Results

### Participant demographic characteristics

Four hundred and eighty children/adolescents along with their parents participated in the study. There were 55.62% boys, 44.37% girls. The M (SD) age was 10.56 (3.89). The majority of the children (95.4%) lived with two parents, mother and father. The evaluation of the economic status of the family based on the FAS indicated that 54.6% of the families belonged to the middle class and 6.7% were among privileged families. There were significant differences according to the child age, mother‘s occupation, and family socio-economic status (Table 1).

**Table 1:** Demographic characteristics of children

	All	Boy	Girl	P
	*N* = 480	*N* = 267 (55.62)	*N* = 213 (44.37)	
	N(%)	N(%)	N(%)	
Child age(year)				0.001^a^
2–4	28 (5.8)	18 (6.7)	10 (4.7)	
5–11	250 (52.1)	117 (43.8)	133 (62.4)	
12 above	202 (42.1)	132 (49.4)	70 (32.9)	
M(SD)	10.56 (3.89)	11.12 (4.23)	9.86 (3.31)	
Grade				0.001^a^
Kindergarten	80 (16.7)	43 (16.1)	37 (17.4)	
Primary	195 (40.6)	87 (32.6)	108 (50.7)	
High school1	133 (27.7)	82 (30.7)	51 (23.9)	
High school2	72 (15.0)	55 (20.6)	17 (8.0)	
Status of living				0.001^a^
Both parent	455 (94.8)	252 (94.4)	203 (96.7)	
Single parent	22 (4.6)	15 (5.6)	7 (3.3)	
Mother age (year)				0.001^a^
34	187 (39.0)	95 (35.6)	92 (44)	
35–44	205 (42.7)	105 (93.3)	100 (47.8)	
45–54	68 (14.3)	54 (20.3)	14 (6.7)	
55 above	16 (3.3)	13 (4.9)	3 (1.4)	
M(SD)	36.92 (8.20)	38.19 (8.73)	35.30 (7.18)	
Mother Education (year)				0.114^a^
0–11	179 (37.3)	97 (36.3)	82 (38.5)	
12	170 (35.4)	99 (37.1)	71 (33.3)	
13–14	60 (12.3)	39 (14.6)	21 (9.9)	
15 and above	71 (14.8)	32 (12.0)	39 (18.3)	
Mother job				0.001^a^
Employed	70 (14.6)	43 (16.1)	27 (12,9)	
Unemployed	407 (84.8)	224 (83.9)	183 (87.1)	
FAS (item)				0.001^a^
Low (8–25)	144 (30.0)	67 (26.9)	77 (40.7)	
Medium(26–42)	262 (54.6)	160 (64.3)	102 (54.0)	
High(43 and above)	32 (6.7.0)	22 (8.8)	10 (5.3)	
M(SD)	29.54 (8.72)	30 (8.0)	28 (8.0)	

*P P*-value, ^a^ *P*-value based on Chi square, ^b^ *P*-value based on t test, M(SD) Mean(Standard Deviation), FAS Family Affluence Scale

### Parent communicative styles

Overall, 12.08% of parents recorded that they did not set regulations to control child sedentary behaviors, 56.45% applied punishment or a threatening style, and 85.2% stated that they used a logical style to communicate and explain reasons. There was a significant difference based on child gender and parent regulation settings and type of the behavior (*P* = 0.001) (Table 2).

**Table 2:** Distribution of M (SD) of Parent behavioral style scores and sedentary behaviors of children/adolescents

	All	Boy	Girl	P
	*N* = 480	*N* = 267 (55.62)	*N* = 213 (44.37)	
Parent behavioral style				
Setting regulations Watching TV				
M(SD)	4.25 (0.88)	4.09 (0.89)	4.14 (0.94)	0.322^b^
Playing computer and video games				
M(SD)	4.20 (0.88)	4.04 (0.934)	4.03 (0.99)	0.096^b^
Communicating electronic media/EMC				
M(SD)	4.22 (0.95)	4.03 (1.00)	4.01 (1.16)	0.042^b^
Threat/punishment Watching TV				
M(SD)	2.58 (1.29)	2.34 (1.14)	2.53 (1.29)	0.001^b^
Playing computer and video games				
M(SD)	2.68 (1.29)	2.47 (1.27)	2.53 (1.31)	0.391^b^
Communicating electronic media/EMC				
M(SD)	2.68 (1.33)	2.51 (1.28)	2.55 (1.31)	0.453^b^
Logical style				
M(SD)	4.20 (0.93)	4.09 (0.96)	4.21 (0.94)	0.967^b^
Playing computer and video games				
M(SD)	4.20 (0.96)	4.04 (0.96)	4.20 (0.97)	0.969^b^
Communicating electronic media/EMC				
M(SD)	4.17 (0.97)	4.09 (0.98)	4.21 (0.94)	0.983^b^
Parent knowledge recommended time				
< 2 year old				< 0.001^a^
Know	233 (48.5)	138 (51.7)	95 (44.6)	
2–4 year old				< 0.001^a^
Know	291 (60.6)	168 (62.9)	123 (57.7)	
5 and above				0.015^a^
Know	308 (64.2)	184 (68.9)	124 (58.2)	

*P P*-value, ^a^ *P*-value based on chi- square, ^b^ *P*-value based on independent t-test, M(SD) Mean(Standard Deviation), TV watching Television watching, EMC Electronic media communication

### Parent knowledge

The percentages of parents who had appropriate knowledge about the recommended amount of sedentary time for their children were reported according to the age groups: (under 2, 2–4, and 5 and above), the correct answers were: 48.5, 60.6, and 64.2% respectively.

There was a significant difference in the level of knowledge according to the child sex (*P* < 0.001, *P* = 0.015) (Table 2).

### Child sedentary behaviors

The M (SD) of total sedentary behavior time on weekdays was 870(331) min/d, or 14.5 h/d. The total time of sedentary behavior excluding night time sleep was 615 (331.6), boys 655 (340) and girls 566 (312) min/d. There were significant differences based on the sex of the child and sedentary behaviors on weekdays and weekend. In all 1.3% of children had lower than 120 min/d sedentary behaviors (Table 3).

**Table 3:** Distribution of frequency and (percentage) of the type of sedentary behaviors among boys and girls

	All	Boy	Girl	P
	*N* = 480	*N* = 267 (55.62)	*N* = 213 (44.37)	
Watching TV (min/d)	N(%)	N(%)	N(%)	0.370^b^
< 120	372 (77.5)	211 (79.0)	161 (75.6)	
120 and over	108 (22.5)	56 (21.0)	52 (24.4)	
Playing computer and video games				< 0.001^b^
< 120	251 (52.4)	121 (45.3)	130 (61.3)	
120 and over	228 (47.6)	146 (54.7)	82 (38.7)	
Communicating electronic media/EMC				0.108^a^
< 120	206 (43.1)	106 (39.8)	100 (47.2)	
120 and over	272 (56.9)	160 (60.2)	112 (52.8)	
Studying duration				0.305^a^
< 120	358 (74.6)	204 (76.4)	154 (72.3)	
120 and over	122 (25.4)	63 (23.6)	59 (27.7)	
All sedentary behaviors				
M(SD)	870 (331.0)	820 (312.0)	909 (340.0)	< 0.001^b^
All sedentary behaviors excluded night time sleep				
M(SD)	615 (331.06)	566 (312.0)	655 (340.0)	< 0.001^b^

*P P*-value, ^a^*P*-value based on Pearson chi-square, ^b^*P*-value based on independent t-test, min/d minute per day, M(SD) Mean(Standard Deviation), TV watching Television watching, EMC Electronic Media Communication

### Watching TV

In all 22.5% of children/adolescents, 24.4% girls, and 21.0% boys spent more than 120 min/d on watching TV. Sex differences were not significant (*P* = 0.370). Child age [OR for 12 year and above =1.37; 95% CI = 0.53–3.54], parent knowledge [OR = 0.59, 95% CI = 0.35–0.99], and communicative styles [OR = 1.43, 95%CI = 1.11–1.86] were predictors of spending more than 120 min/d on watching TV (Table 4).

**Table 4:** Results of logistic regression for watching TV, computer and video gaming times (0–119 = 0, 120 and over = 1)

	Watching TV		Playing computer and video games	
	OR (95%CI)	P	OR(95%CI)	P
Sex				
Girl	Ref. (1)		Ref. (1)	
Boy	1.30 (0.854–1.98)	0.221	2.07 (1.33–3.21)	0.001
Age(year)				
2–4	Ref. (1)		Ref. (1)	
5–11	2.28 (0.90–5.79)	0.081	4.31 (1.21–14.56)	0.024
12 and above	1.37 (0.53–3.54)	0.034	7.44 (2.06–26.77)	0.002
Mother education(year)				
15 and above	Ref. (1)		Ref. (1)	
13–14	0.86 (0.42–1.74)	0.678	1.06 (0.46–2.44)	0.891
12	1.03 (0.52–2.03)	0.931	1.40 (0.68–2.25)	0.353
0–11	0.66 (0.30–1.04)	0.302	1.10 (0.45–1.99)	0.892
Mother job				
Employed	Ref. (1)		Ref. (1)	
Unemployed	1.00 (0.52–1.91)	0.516	0.67 (0.34–1.34)	0.261
FAS(item)				
Low(8–25)	Ref. (1)		Ref. (1)	
Moderate(26–42)	1.38 (0.86–2.21)	0.181	1.88 (1.15–3.07)	0.011
High(43 and above)	1.06 (0.48–2.66)	0.889	1.71 (0.67–4.35)	0.257
Parent behavioral style				
Setting regulations	0.93 (0.713–1.20)	0.572	0.91 (0.71–1.17)	0.482
Threat/punishment	0.97 (0.828–1.13)	0.692	1.14 (0.97–1.33)	0.112
Logical style	1.43 (1.11–1.86)	0.007	0.975 (0.770–1.23)	0.830
Parent knowledge the recommended time				
< 2 year				
know	Ref. (1)		Ref. (1)	
Don’t know	0.592 (0.35–0.99)	0.047	065 (0.38–1.13)	0.126
2-4 year				
know	Ref. (1)		Ref. (1)	
Don’t know	0.74 (0.33–1.61)	0.444	1.41 (0.62–3.19)	0.411
5 year and over				
know	Ref. (1)		Ref. (1)	
Don’t know	0.954 (0.46–1.98)	0.299	0.37 (0.17–0.78)	0.010

*OR* Odds Ratio adjusted for child sex and age, mother job and education level, FAS, parent knowledge, parent behavioral, and communicative styles, *Ref* Reference group, *FAS* Family Affluence Scale

### Playing computer and video games

Forty seven percentage of all children/adolescents, 38.7% girls, and 54.7% boys spent more than 120 min/d on computer and video games playing and the difference between boys and girls was significant (*P* < 0.001). Sex [OR for boys = 2.07, 95% CI =1.33–3.21], age group [OR for 5 and above = 4.31, 95% CI = 1.21–14.56, OR for 12 and above =7.44, 95% CI = 2.06–26.77], family socio-economic status (OR for middle class =1.88, 95% CI = 1.15–3.07], and parent knowledge [OR = 0.37, 95% CI = 0.17–0.78] were predictors of spending more than 120 min/d on computers and video games (Table 4).

### Communicating electronic media/EMC

More than half (56.9%) of all children/adolescents, 52.8% girls, 60.2% boys spent 120 min/d and over on electronic media communication. Sex differences was not significant (*P* = 0.108). Age group [OR for 5 years and above = 4.83, 95% CI =1.52–15.38, OR for 12 years and above = 13.76, 95% CI = 4.22–24.91], family socio-economic status [OR for middle class = 2.52, 95% CI =1.54–4.11, OR for high class 5.53, 95% CI =1.80–15.89], and lack of parent knowledge [OR = 3.03,95% CI = 1.06–8.67] were predictors of spending more than 120 min/d on EMC (Table 5).

**Table 5:** Results of logistic regression for Communicating electronic media/EMC and studying times during on weekdays (0–119 = 0, 120 and over = 1)

	Communicating electronic media/EMC		Studying duration	
	OR (95% CI)	P	OR (95% CI)	P
Sex				
Girl	Ref. (1)		Ref. (1)	
Boy	1.11 (0.71–1.71)	0.677	0.58 (0.35–0.94)	0.028
Age (year)				
2–4	Ref. (1)		Ref. (1)	
5–11	4.83 (1.52–5.38)	0.008	2.27 (0.49–10.63)	0.296
12 and above	3.76 (1.22–4.91)	< 0.001	3.52 (1.19–7.15)	0.029
Mother education (year)				
15 and above	Ref. (1)			
13–14	1.22 (0.61–2.49)	0.582	2.10 (0.83–5.28)	0.113
12	1.10 (0.39–1.81)	0.665	1.36 (0.60–3.88)	0.404
0–11	1.45 (0.62–3.42)	0.385	1.60.67–3.73)	0.285
Mother job				
Employed	Ref. (1)		Ref. (1)	
Unemployed	1.22 (0.68–2.49)	0.582	1.37 (0.64–2.90)	0.411
FAS(item)				
Low (8–25)	Ref. (1)		Ref. (1)	
Moderate (26–42)	2.52 (1.54–4.11)	< 0.001	2.92 (1.58–5.39)	0.001
High(43 and above)	5.53 (1.80–15.89)	0.003	4.17 (1.55–11.28)	0.005
Parent behavioral style				
Setting regulations	7.95 (0.61–1.04)	0.099	1.11 (0.79–1.43)	0.670
Threat/Punishment	1.07 (0.92–1.26)	0.365	1.11 (0.91–1.33)	0.903
Logical style Parent knowledge the recommended time	1.01 (0.7–1.18)	0.482	0.693 (0.533–0.901)	0.006
< 2 year				
Know	Ref. (1)		Ref. (1)	
Don’t know	0.512 (0.28–0.91)	0.922	1.00 (0.54–1.88)	0.988
2–4 year				
Know	Ref. (1)			
Don’t know	1.27 (0.55–2.97)	0.574	3.03 (1.06–8.67)	0.030
5 year and above				
know	Ref. (1)		Ref. (1)	
Don’t know	1.53 (0.69–3.74)	0.305	0.625 (0.23–1.65)	0.343

*EMC* Electronic Media Communication, *OR* Odds Ratio adjusted for child sex and age, mother age, job and education years, FAS, parent knowledge, behavioral style, and parent-child communication, *Ref* Reference group, *FAS* Family Affluence Scale

Eighty six percentage of children (*n* = 28) under 5 years old played on computer, smartphone and other electronic devices more than 30 min/d on weekdays (not indicated on the table).

### Studying duration

About a quarter (25.4%) of children/adolescents, 27.7% girls, 23.6% boys spent 120 and above min/d on studying without any significant differences (*P* = 0.305). Child sex [OR for boys = 0.58, 95% CI = 0.35–0.94], age group [OR for12 year and above = 5.52, 95% CI = 1.19–23.15], socio-economic status [OR for middle class = 2.92; 95% CI =1.58–5.39], [OR for high class = 4.17,95% CI =1.55–11.28], parent knowledge [OR = 3.03, 95% CI =1.06–8.67] were among the main related factors for length of studying time (120 min and more per day) (Table 5).

## Discussion

Prevalence of sedentary behaviors among children /adolescents on weekdays and on the weekend was higher than120 min/d (the ceiling of the recommended time) [6, 12]. There was also a difference in the pattern of sedentary behaviors based on sex and type of sedentary activity.

This study took five major factors of family socio-economic status, parent knowledge and parent communicative styles to assess the patterns of child /adolescent sedentary behaviors. The selected behaviors were watching TV, computer and video gaming, communicating electronic media and studying duration. The level of knowledge of parents regarding the recommended time for child involvement in sedentary behaviors, and parent type of communicative styles and regulation setting indicated significant differences according to the child sex.

The accumulative amount of sedentary behavior time exceeded the recommended time, 120 min/d or 2 h /d [11–14] for boys and girls among Iranian children. Prevalence of sedentary behaviors including working on computers, playing video games and EMC was higher among boys, while the prevalence of watching TV and studying duration was higher among girls.

In general, 22.5% of all children/adolescents, 24.4% girls and 21% boys, watched TV more than 3 h/d; and 28.2% of children/adolescents, 21.2% girls and 33.7% boys, spent more than 3 h/d on computers and video gaming. The prevalence of watching TV more than 3 h/d among Iranian children is similar to that of American [3, 36] and Australian children [5, 37], higher than Spanish [18] and lower than Austria and Welsh children [37]. The prevalence of watching TV more than 4 h /d among American children is 24.7% [4], Australian children 17.21% [5], Spanish (12%) [18], Austria (32–25%), and Wales (36–38%) [38].

The pattern of sedentary behaviors differs according to child sex and socio-economic status of family all over the world [12, 18, 27]. In this study sex differences regarding all kinds of sedentary behaviors including computer and video gaming duration was noticed. Although the prevalence of watching TV and studying duration (≥ 120 min/d) was higher among girls, a significant difference was observed only on studying duration. Boys were high risk gender group in working excessively on computers, video gaming and also EMC, while girls were more interested in watching TV and studying as sitting activities.

Overall, Iranian children (under 5 years old and above) are at risk of spending too much time on working on computers, video gaming and electronic media communications, while 12 year old children and over spend more time (120 min/d) on watching TV and other sitting activities. In general, Iranian children start coming into contact with computer and smartphones from a very young age (toddlers and above). It can be related to the parent perception and attitude [39–41]. Some parents think handling computer and electronic devices sharpens their child‘s intelligence, while others let children play with the device to stay amused and busy and not disturb them.

Both family behavior and the general atmosphere at home combined with environmental factors can make children more vulnerable to sedentary behavior. Parent characteristics, knowledge and their communicative styles and the unhealthy way of controlling children from early childhood play a significant role in the time children spend on sedentary behaviors [18]. In most of the studies a significant correlation was found between parent occupation and educational level [18, 21] and the socio economic status of the families [17], with the sedentary behaviors of the children/ adolescents. The prevalence of child involvement in EMC among wealthy families in western societies has also been observed and documented [42]. However, the present study did not indicate such a correlation; except for the expensive electronic devices such as computers, video gaming and EMC, particularly, among newly emerging well- to-do families in Iran. Iranian children/adolescence of middle class and privileged families are at risk of spending too much time on computers, video games and electronic media communications.

About half of the parents were aware of the optimal or recommended time of sedentary behaviors and applied punishment or adopted threatening styles, while the majority of parents (85.2%) adopted more logical behaviors. Lack of parent knowledge about recommended time for child sedentary behaviors, and unreasonable communicative styles make children/ adolescents attracted to watching TV, playing computer and video games, and other electronic devices.

The relationship between the parent knowledge and behavioral style as cognitive and motivational factors with sedentary behaviors was applicable only on watching TV, while the factors related to computer and video gaming and EMC were restricted merely to the parent knowledge. Excluding watching TV, in this study no significant correlation was shown between child sedentary behaviors and parent behavioral styles. That is, parent-communicative style to motivate children/adolescents to control their excessive sedentary behaviors did not affect any of their sitting behaviors, except watching TV. He et al. also found that parents with a negative attitude towards their children’s sedentary behaviors of more than 2 h/d had set no regulations in this regard [39]. The correlation between parent-child relationship with the time duration of children watching TV was reported [16, 24, 28]. In the study of Schary et al. [25] a meaningful correlation was shown between parent communicative styles and child sedentary activity time. The study revealed that the children of both authoritative and occupied parents spent the minimum time watching TV and playing games on computers [25]. The Bjelland et al. study [27] revealed that the parent supportive style and the child autonomous behavior resulted in minimum time of watching TV and computer gaming among European children. By setting regulations and making children understand the regulations and reasons behind them, autonomy supportive style promotes more mature functioning on behalf of the children [25, 26]. This results in internalization of external stimulation, which in turn, converts the external motivation into internal motivation by children [43, 44].

One of the possible reasons for the lacking of relationship between parent communicative style and computer gaming and electronic media communication times can be related to the general living style among many families in Iran. Although parents set regulations for spending time in sedentary behavior, they probably do not set regulations for other aspects of child life such as outdoor activities, time spent with peer groups, and designated time for going to bed. Therefore, with no alternative for computer and game playing and EMC, and staying awake for long hours before going to bed, it seems not much remains for both child and parent except giving up and breaking the regulations [37]. Additionally, children can be very much attracted to computer gaming and communicating electronic media because these devices provide the kind of privacy and autonomy children typically like to have in their activities far from parents and other adult supervision [44].

### Limitations

The strength of this study was to evaluate children/adolescents sedentary behaviors and the existing correlation between parent knowledge and communicative styles.

However, any kind of physical activities and the child anthropometric measures, especially childhood obesity, were not taken into the consideration. Another weakness goes back to not estimating the pattern of parent sedentary behavior.

Like any other cross-sectional study with a questionnaire as a research instrument, the scales were assessed in a self-reported manner by the participants.

## Conclusion

The prevalence of sedentary behaviors among Iranian children/ adolescents is higher than (120 min/d) recommended. However, the pattern differs according to the type of the activity, age, sex, and the family socio-economic status.

In general Iranian children - especially those who come from middle class and privileged families - start coming into contact with computers, video gaming, and playing on smartphones from a very young age (toddlers and over). There is an urgent need to combat the unrestricted prevalence of sedentary behaviors among Iranian children/ adolescents who use computers and other electronic devices more than the recommended time, especially considering that children are provoked and intrinsically motivated to use these devices because of the feeling of privacy and autonomy they achieve. It is suggested that parent perception and attitude towards children’s handling these devices be considered and corrected by providing suitable educational and instructional programs for the parents.

The adverse side effects of over using electronic devices from very young ages, and appropriate communicative styles in this regards should be taught to the parents through accessible media, health, educational and recreational centers.

## Supplementary information

**Additional file 1.** All raw datasets as supplementary information.

## Data Availability

The dataset supporting the conclusions of this article (Additional file 1) is available at the end of this article.

## Abbreviations

CI: Confidence Interval
EMC: Electronic Media Communication
FAS: Family Affluence Scale
h /d: Hour per day
M (SD): Mean (Standard Deviation)
min/d: Minute per day
OR: Odds Ratio
PC: Personal Computer
STROBE: Strengthening the Reporting of Observational Studies in Epidemiology
TV: Television
